# Commentary: Reproducibility in Psychological Science: When Do Psychological Phenomena Exist?

**DOI:** 10.3389/fpsyg.2017.01004

**Published:** 2017-06-22

**Authors:** Matti T. J. Heino, Eiko I. Fried, Etienne P. LeBel

**Affiliations:** ^1^Department of Social Sciences, University of TampereTampere, Finland; ^2^Department of Social Research, University of HelsinkiHelsinki, Finland; ^3^Department of Psychology, University of AmsterdamAmsterdam, Netherlands; ^4^Department of Psychology, Western UniversityLondon, Canada

**Keywords:** replicability crisis, replication, complexity, complex systems, falsification

[F]alsifiability and replication are of secondary importance to advancement of scientific fields.

—Iso-Ahola, 2017

A cultural divide is forming in psychology: while some researchers are skeptical of research practices and standards prior to the current replicability crisis (e.g., Munafò et al., 2017), others have strong faith in the published literature (e.g., Gilbert et al., 2016). This difference of perspectives drives many current debates, and is also visible in a new paper in *Frontiers in Psychology* by Iso-Ahola, titled “Reproducibility in psychological science: When do psychological phenomena exist”, in which the author states that prior findings cannot be all dismissed as flukes “because they were published in the best journals of social psychology” (Iso-Ahola, 2017).

In the piece, Iso-Ahola sets out the idea that experiments are only able to show evidence *for* phenomena, but can never “prove a negative.” This makes it impossible to falsify psychological ideas like the ego depletion effect (Baumeister et al., 1998). He argues that “reproducibility in psychology is unattainable,” and “that psychological phenomena, by their nature, are not fully reproducible” because humans “can be astonishingly simple or irreducibly complex at various times.” Iso-Ahola further claims that researchers have largely focused on isolated indicators—such as effect size and replicability—to determine whether psychological phenomena exist. Contrasting these practices, he proposes 10 criteria to vet psychological phenomena. Instead of attempting to perform exact replications that make little sense since they cannot disprove theories, the author argues that researchers should use conceptual replications to “attempt to establish a phenomenon's boundary conditions”. Iso-Ahola's default position seems to be that all phenomena exist. He puts the burden of proof on the skeptics, and not on those who claim they have identified psychological phenomena, arguing that “nobody has provided a theoretically and logically rigorous rationale and justification why ego depletion as a phenomenon should and would not exist.”

We agree that some of the language around replication could be more precise and that direct replications could be improved in several respects (Klein et al., 2014; LeBel et al., 2017). We disagree, however, with Iso-Ahola's positions that falsifiability is “of secondary importance” to scientific progress, that psychological phenomena are in principle not “fully reproducible[sic]”, or that one should conduct conceptual replications before ascertaining that there really is something to replicate (and the concepts are sufficiently clear; see Lurquin and Miyake, 2017). Falsification is what makes science self-correcting (Popper, 2005; LeBel et al., in press), and replication *is* possible if researchers clearly specify which conditions are crucial for their hypothesis to hold. Risky tests, for example via sufficiently methodologically similar direct replications, are necessary to produce the insight that some of our ideas may well be wrong; in the absence of such rigorous replications, psychology turns into astrology. Such replications are especially important given that modern psychology has seen numerous highly influential findings not replicate when tested via high-powered and transparently executed direct replications (LeBel et al., in press see curated list of unsuccessful replications at https://osf.io/8srcd/). Iso-Ahola, however, maintains that “phenomena's existence should not be defined by any index of reproducibility of findings.” In our opinion, that is an unnecessary lowering of scientific standards. Instead, falsification via replicability tests forces the investigators to consider more fitting measurement or modeling approaches (person-level time series designs, e.g., Molenaar and Campbell, 2009; highly-repeated within-person designs, e.g., Whitsett and Shoda, 2014), better experimental designs, and more sophisticated paradigms.

Now, we should not pretend falsification is easy, especially in psychology. It is challenging to confirm that auxiliary hypotheses—e.g., soundness of measurement and experimental procedures—are reasonable (Meehl, 1990; LeBel and Peters, 2011; Earp and Trafimow, 2015). This is naturally a challenge for original studies, too. On the bright side, approaches to executing direct replications are becoming more sophisticated. For example, CurateScience.org (LeBel et al., 2017) now presents “active sample evidence” including positive controls, manipulation checks, and measurement integrity (e.g., internal consistency estimates) as a way to help confirm sound auxiliaries (for an example of a replication reporting a positive control, see Sanchez et al., 2017). In addition, links to open materials/data are provided to verify the integrity of replications.

Falsification is also difficult because replications may fail due to problems with operationalizations of the focal constructs. Another reason for lack of replicability and generalizability of psychological phenomena that has been largely overlooked in the extant literature is their complexity: psychological processes such as emotions, cognitions, personality characteristics, or mental disorders are highly multi-causal, with thousands of determinants that often have very small effects on behavior. It seems like Iso-Ahola refers to this, when he talks about the “subtle, elusive” nature of psychological phenomena. But the study of complex dynamic systems (Bar-Yam, 1997; Vallacher and Nowak, 2008) has been widely successful in other areas of research in the last decade (e.g., ecology, biology, and physics), and offers a way forward for psychology too. Complex systems can be characterized as webs of interdependent self-organizing parts whose interactions give rise to emergent properties (Borsboom and Cramer, 2013). Some variations of this perspective have already been brought to at least psychopathology (Fried et al., 2017), personality (Mõttus and Allerhand, 2017), intelligence (Van Der Maas et al., 2017), development (Smith and Thelen, 2003), language (Beckner et al., 2009), and public health (Resnicow and Page, 2008).

The consequence of complexity should not be to abandon replicability, but to embrace it—via sophisticated theories, improved study designs and operationalizations, and modern statistical models that adequately account for the heterogeneity and complexity of psychological phenomena. Replicability is the only way to self-correct our understanding of psychological phenomena in a productive and cumulative fashion.

## Author contribution

All authors listed, have made substantial, direct and intellectual contribution to the work, and approved it for publication.

### Conflict of interest statement

The authors declare that the research was conducted in the absence of any commercial or financial relationships that could be construed as a potential conflict of interest.
